# Transforaminal Epidural Autologous Conditioned Serum Injection in the Treatment of Unilateral Lumbar Radicular Pain: A Randomized, Controlled, Double-Blind Clinical Trial: Role of ACS on Lumbar Radicular Pain

**DOI:** 10.31661/gmj.v11i.2324

**Published:** 2022-12-10

**Authors:** Masoud Hashemi, Siroos Moemenzadeh, Mehrdad Taheri, Payman Dadkhah, Sarah Hojjati, Salman Vojdani

**Affiliations:** ^1^ Anesthesiology Research Center, Shahid Beheshti University of Medical Sciences, Tehran, Iran; ^2^ Department of Exercise Education and Sport Sciences, Shiraz Branch, Islamic Azad University, Shiraz, Iran; ^3^ Non-communicable Disease Research Center, Fasa University of Medical Sciences, Fasa, Iran

**Keywords:** Low Back Pain, Intervertebral Disc Degeneration, Interleukin-1 Receptor Antagonist, Autologous Conditioned Serum

## Abstract

**Background:**

Low back pain could related to disc herniation and managed by surgery. Also, less invasive options, including epidural corticosteroid injection, are available; however, it is associated with side effects. This study aimed to evaluate the effectiveness of autologous conditioned serum (ACS) in treating unilateral lumbar radicular pain.

**Materials and Methods:**

In this randomized, controlled, double-blind clinical trial study, a total of 68 patients received the transforaminal epidural injection, 28 patients received ACS, and 30 patients received 40 mg triamcinolone. Under fluoroscopic guidance in anterior-posterior and lateral views, a single injection of ACS or triamcinolone was done via the transforaminal epidural technique. Pain intensity was assessed with a visual analogue scale (VAS) and Oswestry disability index (ODI) at three weeks, three months, and six months.

**Results:**

A significant reduction in pain intensity was observed in patients of two groups. There was no significant difference between the two groups during the three months of the study. At the final evaluation at six months, the ACS group showed superiority over the triamcinolone based on the VAS score (P0.05) and ODI (P=0.007).

**Conclusions:**

ACS therapy is a new effective option in treating lumbar radicular pain due to herniated disc. Since no specific complication has been reported, it can be used as a substitute for corticosteroids in such cases.

## Introduction

Epidemiological studies show that low back pain (LBP) is reported by 90% of patients, and 35-37% of the world population experience a one-month course of this illness [1]. A major cause of LBP is lumbar nerve root compression by the herniated nucleus pulposus [2]. However, recent studies suggest the role of inflammatory edema associated with the degree of herniated disc irritation [1][2]. In addition to the mechanical component, the biomechanical component also causes pain [3]. The nucleus pulposus contains several pain mediators, such as phospholipase A2, nitric oxide, and prostaglandin E [4]. Annulus fibrosus tear leads to leakage of inflammatory mediators into the epidural space. This leakage causes chemical stimulation and sensitization in the nerve root [5].

Presently, treatment options for disc herniation range from non-invasive methods and physiotherapy to surgical removal of the protruded disc. Although surgery can lead to pain relief, it also has a range of complications, including infection, recurrence of herniation, and formation of epidural scars; therefore, less invasive treatments are considered [6]. Discectomy aspiration, laser discectomy, radiofrequency nucleoplasty, intra-disc electrothermal therapy, and peri-ganglionic corticosteroid are examples of less aggressive methods [7]. Similar to conventional discectomy, the mechanism of most of the methods mentioned is removing the pressure from the nerve root [7].

Another treatment is the injection of corticosteroids around the nerve root. Corticosteroids suppress the inflammatory responses of the disc; however, they are some side effects, such as flushing, weight gain, hyperglycemia, Cushing syndrome, glaucoma, bone density reduction, mood changes, gastric ulcers, increased blood pressure, and increased risk of infection and psychosis [8]. Spinal cord infarction is another complication of corticosteroids when used epidurally, especially with particulate steroids; therefore, non-particulate steroids are used, which are less effective [8][9]. Due to the side effects of existing treatments, the need for a less complicated procedure is evident.

As mentioned, due to the direct toxic effects of inflammatory mediators, an anti-inflammatory environment in the vicinity of the damaged nerve root forms the basis for developing new biological treatment modalities. Interleukin-1 (IL-1) is a master cytokine in pain and inflammation of local and systemic disorders; IL-1 inhibitors have recently been considered in the musculoskeletal system and lumbar spine [10]. Several strategies are used to inhibit the biological activity of IL-1, such as its receptor antagonist and the first type of ILs (IL-4, -10, and -13) that reduce its production [10].

Autologous conditioned serum (ACS) has a high concentration of IL-1 receptor antagonists and is implicated as an IL-1 antagonist in reducing biochemical effects in lumbar radiculopathies [1]. The autologous serum also is enriched with high concentrations of factors, such as insulin-like growth factor 1 (IGF-1), transforming growth factor β1 (TGF-β1), and platelet-derived growth factor (PDGF) [1]. In 2003, Meijer et al. introduced the method of ACS preparation [10]. After that, this method was widely used in other countries. Multiplying the natural synthesis of IL-1 receptor antagonists by monocytes in an in vitro condition produces ACS [11]. The activator of this synthesis is a special borosilicate glass coated with chromium oxide [1][11][12]. ACS has been shown to be effective in musculoskeletal disorders such as knee osteoarthritis, which rely on inflammation [10]. To date, limited studies have been performed to evaluate the effectiveness of this method on lumbar radiculopathy. Hence, this study evaluated the effectiveness of ACS in unilateral lumbar radicular pain.

## Materials and Methods

**Figure-1:**
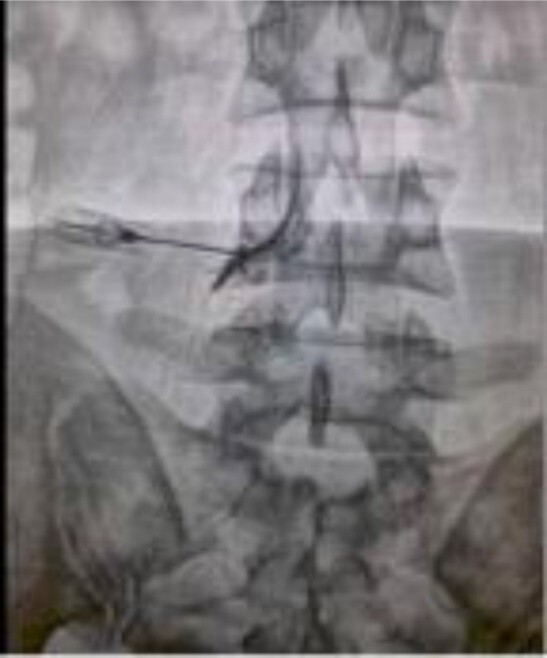
Figure 1. Anterior/posterior radiograph (right-sided) of transforaminal epidural injection at L4-5 intervertebral level.

This randomized, controlled, double-blinded clinical trial study was conducted in an interventional pain management center (Tehran, Iran) from 2019 to 2021 to investigate the effect of transforaminal epidural ACS in the treatment of unilateral lumbar radicular pain.

### Inclusion and Exclusion Criteria

The study enrolled consecutive series of male and female patients aged 18 to 64 years with moderate to severe LBP with radiation to unilateral lower limb due to single-level disc herniation confirmed by magnetic resonance imaging (MRI) of at least six weeks duration and no response to conservative treatments.

The exclusion criteria were severe spinal canal stenosis, systemic bone or joint disease, systemic inflammatory diseases, history of lumbar surgery, concurrent cervical myelopathy, altered coagulation, ongoing infectious disease, steroid injections over the past six months, presence of neurological deficits, need for early surgery, acute trauma, and pregnancy. Patients with missing data were excluded from the study population.

MRI grading was based on Lee et al. [13], and patients with grade 0 (normal), grade 1 (mild foraminal stenosis), and grade 2 (moderate foraminal stenosis) were included in the study. However, patients with grade 3 (severe foraminal stenosis) [13] or central spinal canal stenosis (antero-posterior diameter of the spinal canal<12 mm) were excluded from the study [14].

### Randomization

Based on the eligible criteria, patients were randomly divided into two groups and received transforaminal epidural injections of 4 cc ACS or 40 mg triamcinolone.

### ACS Preparation and Blinding

ACS was prepared as described by Meijer et al. [10]. Briefly, the venous blood was incubated in a special glass containing chromium sulfate. The interaction between cells and glass bead surface increases the production of IL-1 receptor antagonist (IL-1Ra) and anti-inflammatory cytokines. Product centrifugation generated an enriched serum in IL-1Ra and anti-inflammatory cytokines [10]. A physician who evaluated patients after the procedure, as well as patients, were unaware of the allocation of groups.

### Intervention

The patient was in a prone position. Sterile preparation was performed with alcohol and betadine (povidone-iodine) solution. Local anesthesia was provided by injection with 1% lidocaine. The C-arm x-ray device was rotated and tilted to get anterior/posterior (A/P) and oblique views of the target lumbar vertebra. The oblique view was obtained when the superior articular process intersected the center of the pedicle at the target level. The entry point was below the center of the pedicle shadow (6 o'clock). The depth of the 22-gauge Quincke spinal needle was checked with lateral views. The final depth of the needle was located between the middle and the posterior one-third of the intervertebral foramen. An A/P view with 2 mL contrast agent, iohexol-180 (Omnipaque, GE Healthcare, Cork, Ire- land), confirmed the target point (Figure-1). The injected material was 3 cc preservative-free lidocaine 1% plus 40 mg triamcinolone in the first group (control group) and 4 cc ACS in the second one (ACS group). No other medication was added to the ACS group.

### Pain Intensity Measurement

Before the injection, patients documented their pain intensity using the 100 mm Visual Analogue Scale (VAS), ranging from 0 (pain-free) to 100 (greatest pain intensity). Another assessment was the Oswestry Disability Index (ODI). Patients were monitored for 30 minutes after the procedure. Follow-up examinations were scheduled at 3, 12, and 24 weeks following the injection.

### Ethical Considerations

Patients provided written informed consent. All pain medications were discontinued at baseline, and only meloxicam was allowed for pain relief during the study. Patients received no additional treatment. The local ethics committee of Shahid Beheshti University of Medical Sciences approved the study protocol (approval No.: IR.SBMU.REC.1398.034). Also, the study was registered in the Iranian Registry of Clinical Trial (number: IRCT20190513043580N1) and was performed by the ethical standards of the 1964 Declaration of Helsinki.

### Statistical Analysis

The results were given in mean and standard deviation (SD). The Shapiro-Wilk test was used to assess the normality of the data distribution. The independent-sample t-test was applied to compare the differences between groups, and repeated measure analysis followed by the Bonferroni post-hoc test was performed to identify the differences among stages. The Mann-Whitney U test was carried out to compare the satisfaction factor and gender between groups. A P-value of ≤0.05 was considered as statistically significant. All statistical analyses were performed using SPSS software (version 16.0, Chicago, IL, USA).

## Results

**Figure-2:**
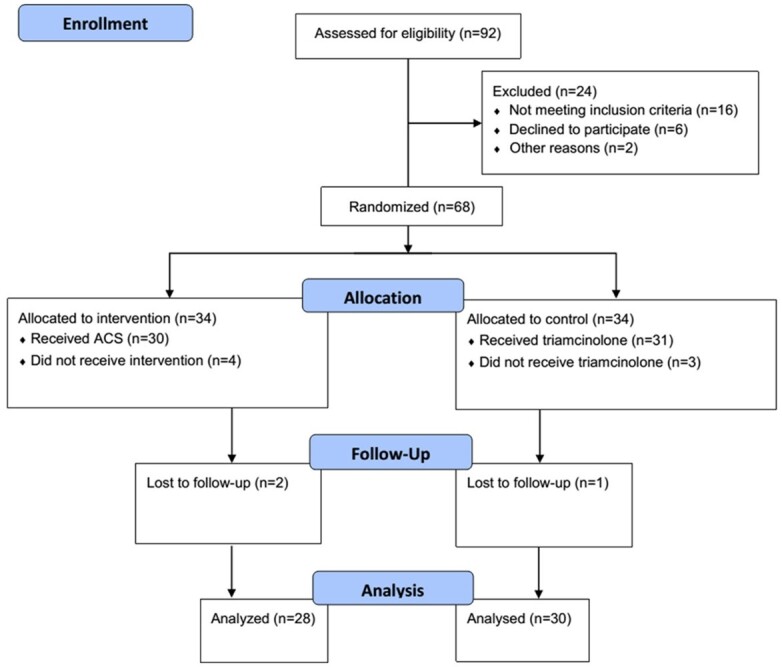
Figure 2. CONSORT flow diagram of the study.

**Table:** Table-1. Demographic Characteristics of Patients.

**Variables**	**Group**		**P-Value**
	**ACS (n=28)**	**Control (n=30)**	
**Age, y**	53.6±7.69	53.96±8.16	0.83
**Height, cm**	163.07±6.9	162.47±8.29	0.76
**Weight, Kg**	75.71±11.44	72.90±10.36	0.33
**Gender**			
Male, n (%)	10 (35.7%)	8 (26.7%)	0.46
Female, n (%)	18 (64.3%)	22 (73.3%)	
*Comorbidity			
Yes, n (%)	10 (35.7)	9 (30)	0.64
No, n (%)	18 (64.3)	21 (70)	
**Disc level**			
L1/L2, n (%)	2 (7.1)	1 (3.3)	
L2/L3, n (%)	2 (7.1)	3 (10)	
L3/L4, n (%)	4 (14.3)	5 (16.7)	0.93
L4/L5, n (%)	12 (42.9)	14 (46.7)	
L5/S1, n (%)	8 (28.6)	7 (23.3)	

**ACS:** Autologous conditioned serum ^*^ Include diabetes, hypertension, and ischemic heart disease

**Table:** Table-2. Pain Intensity Scores Based on VAS

**Groups**		**VAS Score**		
	**Baseline**	**3 weeks**	**12 weeks**	**24 weeks**
**ACS**	7.96±1.23	1.92±1.33	2.17±1.61	2.71±1.48
**Control**	7.93±1.22	2.56±1.16	2.86±1.38	3.63±1.58
**P-value**	0.92	0.057	0.086	0.027

Data are presented as mean±SD. **VAS:** Visual analogue scale; **ACS:** Autologous conditioned serum

**Table:** Table3. Oswestry Disability Index Score

**Groups**		**ODI Score**		
	**Baseline**	**3 weeks**	**12 weeks**	**24 weeks**
**ACS**	42.5±8.82	8±4.48	10.28±4.37	11.21±3.74
**Control**	45±12.86	9.4±3.6	12.53±5.3	15.4±5.99
**P-value**	0.66	0.1	0.14	0.007

Data are presented as mean±SD. **ODI:** Oswestry disability index; **ACS:** Autologous conditioned serum

### Patients Characteristics

As mentioned in Figure-2, out of 92 patients, 34 were excluded from the study population due to not meeting inclusion criteria (16 patients), declining to participate (6 patients), missing data (10 patients), or inability to communicate (2 patients). The demographic characterization of the two groups is presented in Table-1.

### Pain Intensity Based on VAS Score

An independent-sample t-test was conducted to compare the effect of ACS and triamcinolone on pain intensity in four stages: pre-injection (baseline), first (3 weeks), second (12 weeks), and third (24 weeks) follow-ups.

Regarding Table-2, there was no significant difference in the baseline, 3 and 12 weeks between groups (P=0.92, P=0.057, and P=0.086, respectively). However, a significant difference in 24 weeks was observed (P=0.027).

The repeated measure test was performed and followed by a Bonferroni post-hoc analysis to compare the four stages in ACS or control groups. In the ACS group, significant decreases were observed at all follow-up times compared with the baseline (P<0.001, P<0.001, and P<0.001, respectively). A significant increase also was observed at 24 weeks compared to 3 weeks (P=0.042).

Also, in the control group, significant decreases were seen at all the follow-ups compared with the baseline (P<0.001, P<0.001, and P<0.001, respectively). There was a significant increase at 24 weeks compared to 3 and 12 weeks (P=0.001 and P=0.005, respectively).

### The ODI Scores

A Mann-Whitney U test was carried out to compare ODI scores between ACS and control groups. At the baseline, the ODI was greater for the control group than the ACS group (P=0.66, Table-3). There was no significant statistical difference in ODI between the groups at 3 and 12 weeks. However, at 24 weeks, the ACS group had a significant decrease compared to the control group(P=0.007, Table-3). There were no severe complications, such as fever, infection, hematoma, or other major adverse events. One patient from the ACS group complained of a headache after the procedure. This complication was attributed to the transforaminal procedure.

## Discussion

The present study compared the effectiveness of lumbar transforaminal epidural injection of ACS and triamcinolone in patients with unilateral radicular pain due to a single-level lumbar intervertebral disc herniation. Regarding the efficacy of ACS, both the primary and the secondary endpoints (VAS and ODI) showed a statistically significant reduction in pain and disability compared to the baseline. Transforaminal epidural triamcinolone also reduced pain and disability significantly. There was no statistically significant difference between the two groups during the three-month follow-ups. In the 24^th^ week, ACS showed superiority over the control group in term of the VAS score.

There was a statistically significant difference between the two groups regarding ODI at the end of the study; the ACS group had a significant decrease compared to the control group. To date, there are limited data to evaluate the efficacy of treating lumbar radicular pain with ACS. Kumar *et al.* [3] used ACS for the treatment of lumbar radiculopathy with 2 cc autologous blood serum injection up to a maximum of three times at 7-day intervals based on the patient's clinical response. They evaluated patients by VAS and ODI before and after peripheral epidural injection at two weeks, one month, and six months. In line with our findings, their results showed significant changes in all parameters at all measured intervals and a little tendency to worsen the VAS score with ACS [3]. In our study, there was a single injection of ACS or triamcinolone, whereas, in Kumar *et al*. study [3], patients received two injections of triamcinolone on average. Further, the present study had a control group and included more patients [3].

A pilot study [1] was conducted in 2016 in which 15 patients with single-level disc herniation received six doses of ACS in the intervertebral foramen. Patients were assessed at one and three months after the last dose. Two of the 15 patients underwent surgery due to increased pain. Indeed, those patients had a disc size greater than 8 mm [1]. In the present study, patients with spinal canal stenosis were excluded. However, similar to our study, there was significant pain relief in Godek study [1].

In Becker *et al*. study [2], 32 patients received ACS, 27 patients received 5mg triamcinolone, and 25 patients received 10mg triamcinolone. Injections were done for three consecutive weeks. Similar to our findings, in a six-month follow-up, all patients showed a significant reduction in pain and disability, and also, the results of the ACS group were superior to the other two groups; in long-term pain relief, ACS was superior to triamcinolone [2].

In addition to the anti-inflammatory effects of ACS due to the IL-1Ra, several growth factors such as PDGF, IGF-1, and TGF-β1 have restorative and healing effects [12].

Studies have shown that the anti-inflammatory effects alone are not enough to treat back pain and disability, and the addition of growth factors plays an essential role in relieving pain and disability [3]. For these reasons, ACS is superior to corticosteroids in treating radiculopathies and can reduce the side effects of corticosteroids [3]. As an autologous serum, ACS had no side effects [12]. The transforaminal epidural injection had rare complications, mainly secondary to inadvertent intravascular injection. To detect inadvertent intravascular injection, digital subtraction fluoroscopic imaging is more accurate than blood aspiration and live fluoroscopy [13]. The average depth to the epidural space in the transformational approach in individuals weighing 60 to 70 kg is 6.42 cm, and predicting this space plays an important role in performing this procedure properly and reducing its complications [15]. In the current research, one patient in the ACS group had a post-procedural headache. An inadvertent dural puncture can cause a headache. This adverse effect is attributed to the transforaminal technique [16].

One of the limitations of this study was the lack of post-procedure MRI; the inclusion of radiologic investigations in future studies can overcome this problem. Also, due to the irregular use of meloxicam and lack of remembering meloxicam doses in patients, it was not possible to calculate the use of meloxicam during the study.

Another factor that can affect ACS consumption is its relatively high cost and needing more time to prepare against corticosteroid injections, and in most cases, it is not covered by insurance. However, ACS is considered a novel treatment without any significant side effects, with a more potent and longer-lasting effect than corticosteroids.

## Conclusion

The present study indicates that ACS therapy was a new effective alternative in the treatment of lumbar radicular pain due to herniated discs. Since no specific complication has been reported, it can be used as a substitute for corticosteroids in these cases. Further evaluations for the long-term effects of ACS are suggested.

## Conflict of Interest

The Authors declare that there is no conflict of interest.
